# Suicide mortality trends by sex, age and method in Taiwan, 1971–2005

**DOI:** 10.1186/1471-2458-8-6

**Published:** 2008-01-08

**Authors:** Jin-Jia Lin, Tsung-Hsueh Lu

**Affiliations:** 1 Department of Psychiatry, Chi-Mei Medical Center, Tainan, Taiwan; 2 Institute of Public Health, College of Medicine, National Cheng Kung University, No 1, Dah Hsueh Road, Tainan 701, Taiwan

## Abstract

**Background:**

Method-specific suicide trends varied across countries, and studies of the trends in different countries can contribute to the understanding of the epidemiology of suicide. The purpose of this study was to examine the changes in suicide trends by sex, age and method in the years 1971 to 2005 in Taiwan.

**Methods:**

Mortality data files of suicide and undetermined deaths for the years 1971–2005 were obtained for analyses. Age-, sex- and method-specific suicide rates were calculated by four age groups (15–24, 25–44, 45–64 and 65 and above) and five suicide methods (solids/liquids poisoning, other gases poisoning, hanging, jumping, and others).

**Results:**

Both sexes experienced downward trends from 1971 to 1993, and then an upward trend since 1993. People aged 65 years and above had the highest suicide rates throughout the study periods. However, males aged 25–64 years experienced the steepest increasing trends. As to suicide methods, an annual increase, since 1991, of people jumping from heights to commit suicide, and a marked increase, since 1998, of people completing suicide by poisoning with other gases (mainly charcoal-burning) were observed.

**Conclusion:**

Suicide by means of charcoal-burning and jumping from heights has become a serious public health problem in Taiwan. Preventive measures to curb these increasing trends are urgently needed.

## Background

Suicide is an important public health problem throughout the world. Approximately one million people committed suicide in 2000 [1]. In the last 50 years, suicide rates have increased by 60% worldwide [1]. The suicide trends in most studied countries have been stable or decreasing for females, while the trends for males, particularly the younger age groups, have been increasing [2]. Worldwide suicide trends showed a substantial rise among younger people, i.e., the proportion of those aged 5–44 years committing suicide rose from 40% in 1950 to 55% in 2000 [3]. In addition, the percentages of different suicide methods used have also changed across time in different countries [4-6], partly due to differential availability and sociocultural acceptability of suicide methods [7].

However, most of the previous studies on suicide trends and suicide methods were based on data from Western countries. Data from non-Western countries, such as Asian countries, might illustrate different trends. For example, in Hong Kong, suicide rates were on the increase among the young and the old in 1981–1994 and jumping from heights was the most favored method [8]. In China, a decreasing trend in youth suicide was found in 1991–2000 [9] and pesticide poisoning was very common in China's rural areas [10]. So, knowledge of suicide trends and changes in suicide methods in different countries can contribute to the understanding of the epidemiology of suicide.

Taiwan, a country with 23 million people, has experienced rapid economic growth and industrialization during the past 30 years, and a gradual liberalization of social and political restraints in the most recent decade. A resurgence in suicide trends was found in the late 1990s [11], and suicide became the ninth leading cause of death in the seven years from 1999 to 2005 in Taiwan [12]. Nevertheless, little is known about the details of the demographic and method profiles of this increase.

The aim of this study was to examine changes in suicide trends by sex, age and use of specific methods among defined subgroups during the periods between 1971 and 2005 in Taiwan, especially focusing on contemporary trends.

## Methods

### Data sources

Electronic mortality data files of those aged 15 years and above were provided by the Department of Health of the Executive Yuan of Taiwan for the years 1971–2005.

Since suicide mortality statistics are usually under-estimated, and the most commonly misclassified category is death from undetermined causes [13,14], suicide deaths were defined as those coded E950-E959 and E980-E989 according to the International Classification of Diseases (ICD) in this study. In Taiwan, the ICD-8 was used for the years 1971–1980, and the ICD-9 was used for the years 1981–2005. The ICD codes for suicide did not change from the Eighth Revision to the Ninth Revision. Mid-year populations were obtained from the Demographic Yearbook published by the Ministry of the Interior, Taiwan.

### Data analysis

Sex-, age- and method-specific death rates were calculated to examine suicide trends. Age-adjusted suicide rates were calculated using the world population structure as a standard. Victim age was divided into four groups: 15–24, 25–44, 45–64, and 65 years and older. The different suicide methods were grouped into five categories: solids/liquids poisoning (E950 and E980), poisoning by other gases (E952 and E982), hanging (E953 and E983), jumping from heights (E957 and E987) and other methods (E951, E954-E956, E958-E959, E981, E984-E986, and E988-E989).

For further analysis of the method-specific suicide trends in sex- and age-specific subgroups in the contemporary years of 1991–2005, we combined 3 years of data to minimize the effect of yearly fluctuations.

## Results

### General trend

**Figure 1:**
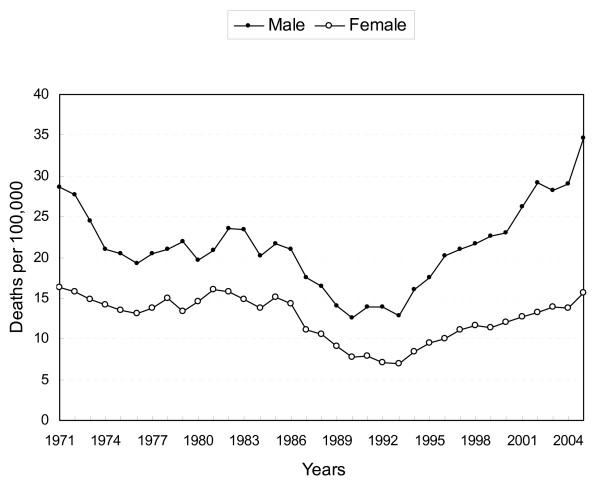
Age-adjusted suicide death rates by sex in Taiwan, 1971–2005.

A V-shape suicide trend was noted from 1971 to 2005. Both sexes experienced downward trends from 1971 to 1993, and then an upward trend from 1993 (Figure 1). A more than 2.7-fold increase in suicide rates was noted for males, from 12.8 per 100,000 in 1993 to 34.6 in 2005. For females, a 2.3-fold increase was found, from 6.9 in 1993 to 15.7 in 2005. The sex ratio of suicide rates also increased, from 1.9 (12.8/6.9) in 1993 to 2.2 (34.6/15.7) in 2005.

### Trend by age

**Figure 2:**
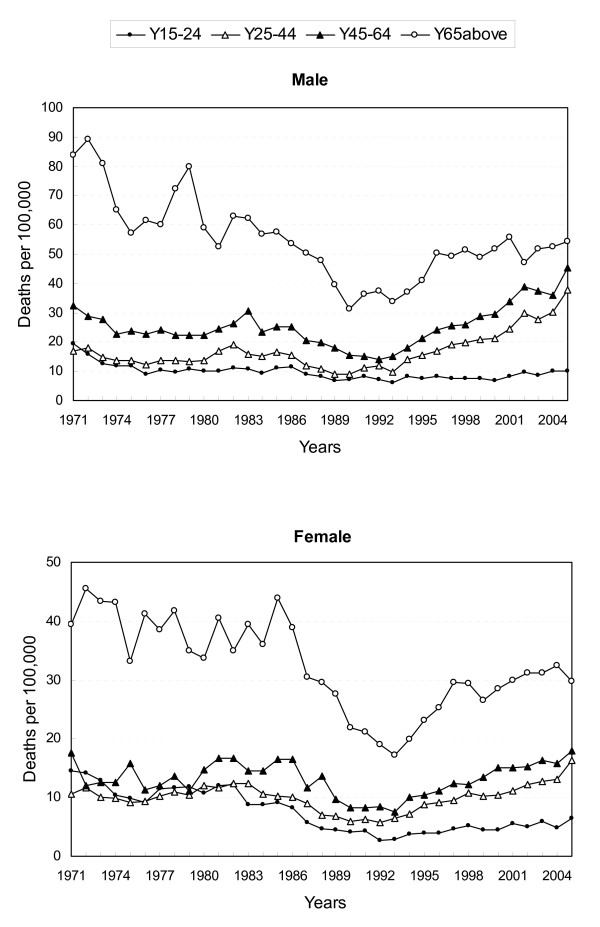
Age-adjusted suicide death rates by sex and age in Taiwan, 1971–2005.

Figure 2 shows the changes in suicide rates by age. People aged 65 and above had the highest suicide rates throughout the study periods. Compared with other age groups, young and middle-aged adults (aged 25–64 years) experienced the steepest increasing trends from 1993 through 2005 in both sexes. The suicide rate for males aged 45–64 was 15.2 per 100,000 in 1993 and 45.2 in 2005, a three-fold increase; for females aged 45–64, there was a 2.4-fold increase (from 7.5 per 100,000 in 1993 to 18.0 in 2005). For males aged 25–44, the rate was 9.7 per 100,000 in 1993, and 37.6 in 2005, an almost four-fold increase; for females aged 25–44, there was a 2.5-fold increase (from 6.4 per 100,000 in 1993 to 16.2 in 2005).

### Trend by method

**Figure 3:**
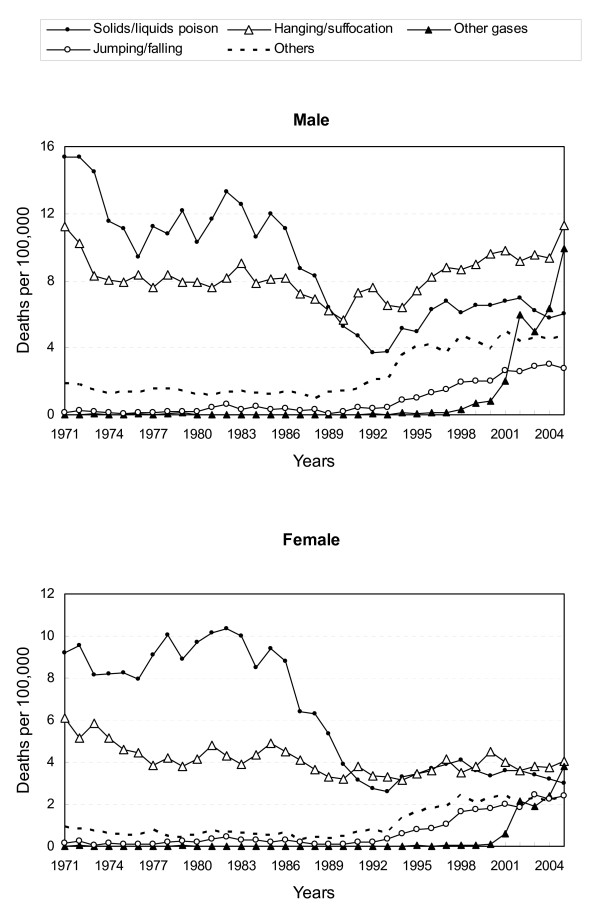
Age-adjusted suicide death rates by sex and method in Taiwan, 1971–2005.

During the 1970's and 1980's, the most commonly used suicide method was poisoning by solids/liquids (Figure 3). A marked decrease in solids/liquids poisoning suicide rates was noted during the 1980s. However, the suicide rates for hanging were relatively stable throughout the study period. Since the 1990s, hanging has surpassed solids/liquids poisoning as the most often used method. These two methods were responsible for about 90% of all suicide deaths before 1990, downward to two-thirds in 2000, and even a half in 2005. While other methods, such as jumping from heights and poisoning by other gases accounted for more suicide deaths in the most recent decade than they did before 1990 in Taiwan.

In 1991, only 47 people completed suicide by jumping from heights; this number increased to 502 in 2005. The age-adjusted suicide rate for jumping from heights increased 6.8-fold in males (from 0.41 per 100,000 in 1991 to 2.78 in 2005), and 13-fold in females (from 0.18 per 100,000 in 1991 to 2.40 in 2005) in the most recent 15 years.

In 1998, only 32 people completed suicide by poisoning with other gases. However, more than 1300 people used this method to kill themselves in 2005. The age-adjusted suicide rate of poisoning with other gases increased 33-fold in males (0.30 per 100,000 in 1998 to 9.89 in 2005), and 126-fold in females (0.03 per 100,000 in 1998 to 3.79 in 2005), an astonishing upsurge.

In the contemporary years of 1991–2005, nearly all method-specific suicide rates seemed to increase in the four age groups in both sexes (Tables 1 and 2). However, the distribution of suicide methods changed much. The weight of the traditional suicide methods in Taiwan (i.e., hanging and poisoning by solids/liquids) decreased, while that of the new methods (i.e., jumping from heights and poisoning with other gases) markedly increased. In 2003–2005, Jumping from heights became the most common suicide method for females aged 15–24 years. Poisoning with other gases became the most common method of suicide deaths in those aged 25–44 years in both sexes and males aged 15–24 years.

**Table 1:** The numbers (N), rate (deaths per 100,000) and percentage (%) of male suicide deaths by method in Taiwan, 1991–2005

	1991–1993		1994–1996		1997–1999		2000–2002		2003–2005	
	
Age	N	Rate (%)	N	Rate (%)	N	Rate (%)	N	Rate (%)	N	Rate (%)
15–24										
Poisoning^a^	144	2.5 (34)	128	2.2 (27)	104	1.7 (23)	93	1.5 (18)	67	1.1 (12)
Hanging^b^	178	3.0 (42)	138	2.4 (29)	154	2.6 (34)	170	2.8 (35)	155	2.7 (28)
Gases^c^	0	0.0 (0)	4	0.1 (1)	6	0.1 (1)	59	0.9 (11)	176	3.0 (31)
Jumping^d^	15	0.3 (4)	56	1.0 (12)	60	1.0 (13)	71	1.2 (15)	93	1.6 (17)
Others^e^	87	1.5 (20)	146	2.5 (31)	130	2.2 (29)	105	1.7 (21)	68	1.2 (12)
25–44										
Poisoning^a^	390	3.7 (34)	573	5.1 (33)	677	5.9 (30)	672	5.9 (23)	624	5.3 (17)
Hanging^b^	539	5.1 (47)	612	5.4 (35)	849	7.4 (37)	882	7.6 (30)	965	8.3 (26)
Gases^c^	5	0.1 (0)	20	0.2 (1)	59	0.5 (3)	564	4.9 (20)	1288	1.2 (35)
Jumping^d^	35	0.3 (3)	109	1.0 (6)	212	1.9 (9)	267	2.3 (9)	339	3.0 (9)
Others^e^	185	1.7 (16)	412	3.7 (24)	482	4.2 (21)	513	4.4 (18)	481	4.1 (13)
45–64										
Poisoning^a^	244	4.8 (32)	391	7.4 (35)	543	9.4 (35)	659	10.2 (30)	651	9.1 (23)
Hanging^b^	398	7.6 (52)	468	8.8 (42)	611	10.5 (39)	823	12.7 (37)	1031	1.1 (35)
Gases^c^	1	0.0 (0)	3	0.1 (0)	35	0.6 (2)	211	3.0 (9)	561	7.1 (18)
Jumping^d^	23	0.4 (3)	48	0.9 (4)	93	1.6 (6)	159	2.4 (7)	224	3.0 (8)
Others^e^	98	1.9 (13)	205	3.9 (19)	271	4.7 (18)	394	5.9 (17)	466	6.4 (16)
65 and above										
Poisoning^a^	175	7.4 (21)	251	9.6 (22)	342	11.8 (24)	401	13.4 (26)	386	1.0 (23)
Hanging^b^	520	23.4 (65)	587	22.6 (53)	724	24.9 (50)	778	24.2 (47)	831	2.0 (45)
Gases^c^	0	0.0 (0)	0	0.0 (0)	2	0.1 (0)	17	0.6 (1)	43	1.4 (3)
Jumping^d^	24	1.1 (3)	64	2.3 (5)	133	4.4 (9)	201	5.9 (12)	199	5.4 (10)
Others^e^	88	3.9 (11)	221	8.4 (20)	249	8.6 (17)	238	7.3 (14)	349	1.1 (19)
Total										
Poisoning^a^	953	4.0 (30)	1343	5.5 (30)	1666	6.4 (30)	1825	6.8 (26)	1728	6.0 (20)
Hanging^b^	1635	7.1 (53)	1805	7.3 (41)	2338	8.8 (40)	2653	9.5 (36)	2982	1.1 (33)
Gases^c^	6	0.0 (0)	27	0.1 (1)	102	0.4 (2)	851	3.0 (11)	2068	7.1 (23)
Jumping^d^	97	0.4 (3)	277	1.1 (6)	498	1.8 (8)	698	2.4 (9)	855	2.9 (9)
Others^e^	458	1.9 (14)	984	4.0 (22)	1132	4.3 (20)	1250	4.5 (17)	1364	4.6 (15)

^a ^Poisoning by solid or liquid substances.

^b ^Hanging, strangulation and suffocation.

^c ^Poisoning by other gases.

^d ^Jumping/falling from high places.

^e ^Others including poisoning by domestic gases, drowning, firearms, cutting and piercing, other and unspecified means, and late effects of injury.

**Table 2:** The numbers (N), rate (deaths per 100,000) and percentage (%) of female suicide deaths by method in Taiwan, 1991–2005

	1991–1993		1994–1996		1997–1999		2000–2002		2003–2005	
	
Age	N	Rate (%)	N	Rate (%)	N	Rate (%)	N	Rate (%)	N	Rate (%)
15–24										
Poisoning^a^	77	1.4 (42)	80	1.4 (37)	63	1.1 (23)	50	0.9 (17)	39	0.7 (13)
Hanging^b^	72	1.3 (39)	57	1.0 (26)	78	1.3 (28)	64	1.1 (22)	76	1.4 (24)
Gases^c^	0	0.0 (0)	0	0.0 (0)	2	0.0 (1)	26	0.4 (9)	77	1.4 (24)
Jumping^d^	13	0.2 (7)	34	0.6 (16)	90	1.6 (33)	94	1.6 (33)	87	1.6 (28)
Others^e^	22	0.4 (12)	44	0.8 (20)	42	0.7 (15)	54	0.9 (19)	36	0.6 (11)
25–44										
Poisoning^a^	265	2.6 (43)	351	3.2 (39)	311	2.8 (27)	266	2.3 (21)	231	2.0 (14)
Hanging^b^	261	2.5 (41)	286	2.6 (32)	356	3.2 (32)	329	2.9 (26)	316	2.8 (20)
Gases^c^	0	0.0 (0)	7	0.1 (1)	10	0.1 (1)	181	1.6 (15)	511	4.6 (33)
Jumping^d^	31	0.3 (5)	94	0.9 (11)	198	1.8 (18)	211	2.0 (18)	300	2.7 (19)
Others^e^	72	0.7 (12)	161	1.5 (18)	249	2.3 (22)	261	2.3 (21)	222	2.0 (14)
45–64										
Poisoning^a^	162	3.4 (42)	209	4.0 (38)	303	5.3 (42)	301	4.6 (31)	322	4.4 (26)
Hanging^b^	178	3.7 (46)	214	4.1 (39)	238	4.0 (32)	344	5.2 (34)	372	5.0 (30)
Gases^c^	0	0.0 (0)	0	0.0 (0)	1	0.0 (0)	58	0.8 (5)	179	2.2 (13)
Jumping^d^	10	0.2 (3)	30	0.6 (5)	60	1.0 (8)	103	1.5 (10)	160	2.1 (13)
Others^e^	34	0.7 (9)	97	1.9 (18)	138	2.3 (18)	195	2.9 (19)	220	3.0 (18)
65 and above										
Poisoning^a^	111	5.6 (29)	175	7.8 (34)	279	10.9 (38)	334	11.3 (38)	340	1.4 (33)
Hanging^b^	234	11.7 (61)	235	10.2 (45)	292	11.2 (39)	352	11.8 (39)	362	1.7 (34)
Gases^c^	0	0.0 (0)	2	0.1 (0)	0	0.0 (0)	4	0.1 (0)	12	0.4 (1)
Jumping^d^	2	0.1 (1)	25	1.1 (5)	41	1.5 (5)	93	3.1 (10)	119	3.6 (11)
Others^e^	32	1.7 (9)	80	3.5 (15)	124	4.8 (17)	107	3.6 (12)	202	6.0 (19)
Total										
Poisoning^a^	615	2.8 (39)	815	3.5 (37)	956	3.9 (34)	951	3.5 (28)	932	3.2 (22)
Hanging^b^	745	3.5 (48)	792	3.4 (37)	964	3.8 (33)	1089	4.0 (32)	1126	3.9 (27)
Gases^c^	0	0.0 (0)	9	0.0 (0)	13	0.1 (0)	269	1.0 (8)	779	2.7 (19)
Jumping^d^	56	0.2 (3)	183	0.8 (8)	389	1.5 (13)	501	1.9 (15)	666	2.4 (16)
Others^e^	160	0.7 (10)	382	1.6 (18)	553	2.2 (19)	617	2.3 (18)	680	2.3 (16)

^a ^Poisoning by solid or liquid substances.

^b^Hanging, strangulation and suffocation.

^c ^Poisoning by other gases.

^d^Jumping/falling from high places.

^e^Others including poisoning by domestic gases, drowning, firearms, cutting and piercing, other and unspecified means, and late effects of injury.

## Discussion

### Changes in suicide rates by sex

Our findings indicate a dramatic fall and rise in the pattern of suicide trends in Taiwan from 1971 through 2005, which was quite different from those in Western countries. Most Western countries showed stable or mildly decreasing trends during the 1980s and 1990s [2,15,16]. However, a similar pattern of increasing suicides since the mid-1990s was also found in Japan [17]. One speculation regarding the cause of the increase was the economic crisis occurring around the same time [11,17,18].

With regard to the male/female suicide ratio, Chinese societies (e.g., Hong Kong and China) had a relatively lower sex ratio than Western countries [19] The male-to-female ratio in Taiwan was higher than those in Hong Kong and China. In contrast to the stable male/female sex ratio in Hong Kong [8], Taiwan showed a small increase in the male-to-female ratio during the past decade, similar to those in China [9]. However, the increase in the male-to-female ratio in Taiwan was owever, driven by the relatively greater increase in male suicide rates, while in China the increase was due to the relatively greater decrease in female suicide rates [20].

### Changes in suicide rates by age

Marked age differences in suicide trends have been noted in Taiwan in the recent decade. A prominent increase in suicide rates occurred among those aged 25–64 years old, particularly males. The continuing increase of annual unemployed rates in the middle and late 1990s in Taiwan [11] might also partly explain why men aged 25–64 had the most prominent increase in suicide rates since 1993, and why men contributed more than women to the increase in the overall mortality trend, since men of this age group were the main income earners in the household, and suffered the most during the economic recessions.

In some Western countries, such as Scotland [21] and New Zealand [4], the suicide rates among adolescents and young adults increased rapidly compared with other age groups. However, this was not the case in Taiwan, where we found only a small increase in suicide rates among men aged 15–24 since 2000. The onset of the increase in suicide rates among women aged 15–24 was earlier, beginning in 1992. Further studies are needed to explore the reasons for these international differences.

### Changes in suicide rates by method

Methods of suicide differed across countries [6]. For example, jumping from heights was the most favored method in Hong Kong, where 85% of the people live in tall buildings [8]. Pesticide poisoning was very common in China's rural areas [10]. In the United States, firearm use accounted for two-thirds of all suicide methods [22]. In Taiwan, as our study revealed, solids/liquids poisoning was the most common suicide method before 1990. Among solids/liquids poisons, pesticides were the most frequently ingested poisons, intentional or unintentional, causing fatality in Taiwan [23]. Our study indicated a significant reduction in solids/liquids suicide rates during the 1980s. Previous study also revealed a prominent decreasing of death rates from solids/liquids poisoning in the same period [24]. The effectiveness of series of countermeasures launched by the Government on solids/liquids suicide rates was assessed in another study [25]. These findings were consistent with a recent systematic review that restricting access to lethal methods could effectively reduce suicide rates [26].

Poisoning by other gases as a method of suicide has become epidemic recently among both sexes aged 25–44 years in Taiwan. Among the other gases suicide deaths, at least 60% of them were charcoal burning in Taiwan [27]. It is highly possible that this new trend was due to widespread coverage in the media of charcoal burning method [28], a copycat effect of media coverage on suicide as supported by several previous studies. [29-31]. Particularly, charcoal burning was reported by the media as an easy, painless, and effective way of suicide and more acceptable in Asian culture [32]. One study in Hong Kong reported that people who completed suicide by inhaling charcoal burning gases were more likely to have been economically active and overly in debt [33]. The 25–44-year age groups in Taiwan seemed to be in concordance with those characteristics. Further studies are needed to determine the reasons for this epidemic occurrence.

Methods of suicide also differed among both sexes. Many studies have indicated that females tended to use drug overdoses and males more frequently used more lethal methods, including hanging, carbon monoxide poisoning, and firearms [34]. However, in Taiwan, hanging was the leading method of suicide among both sexes in the most recent decade. Moreover, suicide by jumping from heights, a violent method, was preferred by younger females, similar to a report from Hong Kong [8], in which people aged below 25 years were prone to adopt jumping as suicide method.

### Methodological limitations

One limitation of using official suicide rates is the underreporting [35]. In Taiwan, a death verdict of unnatural causes is jointly assigned by a prosecutor and coroner, whose main concern is the possibility of homicide. [36] Therefore, a suicide or undetermined verdict helps only in excluding this possibility. However, the intent of this study was to examine the pattern of suicide trends, and it was very unlikely that the artifacts of underreporting would change with time, particularly if undetermined deaths were added into the analysis. Second, the study was descriptive in design, and the delineation of the complex relationships among risk factors of suicide was beyond the scope of this study.

## Conclusion

In conclusion, a substantial rise in suicide rates was found among males aged 25–64 in the most recent decade in Taiwan. Suicide by means of charcoal-burning and jumping from heights has become a serious public health problem in Taiwan. Preventive measures are urgently needed to curb these escalating trends.

## Competing interests

The author(s) declare that they have no competing interests.

## Authors' contributions

JJL contributed to the study design, analysis and interpretation of the data and drafted the paper. THL contributed to the study design, obtained the data and commented on the interpretation. All authors have read and approved the final manuscript.

## Pre-publication history

The pre-publication history for this paper can be accessed here:


